# Influenza Vaccination in Psoriatic Patients—Epidemiology and Patient Perceptions: A German Multicenter Study (Vac-Pso)

**DOI:** 10.3390/vaccines9080843

**Published:** 2021-08-01

**Authors:** Christian Kromer, Phoebe Wellmann, Ralf Siemer, Selina Klein, Johannes Mohr, Andreas Pinter, Dagmar Wilsmann-Theis, Rotraut Mössner

**Affiliations:** 1 Department of Dermatology, University Medical Center Göttingen, 37075 Göttingen, Germany; christian.kromer@med.uni-goettingen.de (C.K.); johannes.mohr@med.uni-goettingen.de (J.M.); 2 Faculty of Medicine, University of Göttingen, 37073 Göttingen, Germany; phoebe.wellmann@stud.uni-goettingen.de; 3 Faculty of Mathematics and Computer Science, University of Göttingen, 37073 Göttingen, Germany; ralf.siemer@stud.uni-goettingen.de; 4 Department of Dermatology and Allergy, University Bonn, 53127 Bonn, Germany; Selina.klein@ukbonn.de (S.K.); dagmar.wilsmann-theis@ukbonn.de (D.W.-T.); 5 Department of Dermatology, Venereology and Allergology, University of Frankfurt, 60590 Frankfurt am Main, Germany; andreas.pinter@kgu.de

**Keywords:** psoriasis, atopic dermatitis, influenza, vaccination, pneumonia

## Abstract

The risk of developing severe complications from an influenza virus infection is increased in patients with chronic inflammatory diseases such as psoriasis (PsO) and atopic dermatitis (AD). However, low influenza vaccination rates have been reported. The aim of this study was to determine vaccination rates in PsO compared to AD patients and explore patient perceptions of vaccination. A multicenter cross-sectional study was performed in 327 and 98 adult patients with PsO and AD, respectively. Data on vaccination, patient and disease characteristics, comorbidity, and patient perceptions was collected with a questionnaire. Medical records and vaccination certificates were reviewed. A total of 49.8% of PsO and 32.7% of AD patients were vaccinated at some point, while in season 2018/2019, 30.9% and 13.3% received an influenza vaccination, respectively. There were 96.6% and 77.6% of PsO and AD patients who had an indication for influenza vaccination due to age, immunosuppressive therapy, comorbidity, occupation, and/or pregnancy. Multivariate regression analysis revealed higher age (*p* < 0.001) and a history of bronchitis (*p* = 0.023) as significant predictors of influenza vaccination in PsO patients. Considering that most patients had an indication for influenza vaccination, the rate of vaccinated patients was inadequately low.

## 1. Introduction

Seasonal influenza is a viral infectious disease, which can be associated with serious morbidity such as bacterial superinfection, influenza encephalitis and myocarditis, hospitalization, and increased mortality [1,2,3]. In particular, older adults, very young children, pregnant women, and patients with certain chronic medical conditions such as chronic heart and lung diseases and metabolic disorders or an impaired immune system, for example, due to immunosuppressive medication, are prone to a more severe course of infection and complications [1,2,3,4,5]. In Germany, the permanent vaccination commission (STIKO) of the Robert Koch Institute (RKI) recommends a yearly influenza vaccination as standard vaccination for all adults ≥60 years of age [6]. The indication group includes, among others, patients with chronic diseases (i.e., respiratory, cardiovascular, liver, kidney, neurological, and metabolic diseases including diabetes) and persons with congenital or acquired immunodeficiency, irrespective of their age [6].

Psoriasis (PsO) is a chronic inflammatory skin disorder with a prevalence of 2–4% in Western countries [7]. In recent years, growing evidence on the immunopathology of the disease has led to the commonly accepted classification as a chronic inflammatory systemic disease associated with a range of comorbidities, including psoriatic arthritis (PsA), obesity, cardiovascular diseases, and metabolic disorders [7]. Moreover, systemic immunosuppressive or immunomodulatory agents, including conventional systemic drugs and biologics, are frequently applied in patients with moderate-to-severe PsO and/or PsA [8,9]. Consequently, the risk for respiratory tract infections, including pneumonia, is considerably increased in PsO patients [10,11,12]. Although diagnosis of PsO alone is currently not explicitly mentioned as an indication for influenza vaccination by the RKI [6,13,14], there are a considerable number of PsO patients who are—irrespective of their age—at increased risk for serious infection due to systemic immunosuppressive or immunomodulatory medication or the above-mentioned comorbidity, and thus should receive yearly influenza vaccinations, following the RKI recommendations [6,13,14]. Influenza vaccination has been studied in other chronic inflammatory diseases such as rheumatoid arthritis [15]. However, there is limited evidence on actual vaccination rates in psoriatic patients in Germany [16]. Therefore, the objective of this study was to determine the rate of seasonal influenza vaccination among PsO patients as compared to patients with atopic dermatitis (AD) and to identify patient perceptions of and potential barriers to vaccination.

## 2. Materials and Methods

### 2.1. Study Design

In this multicenter cohort study, adult patients with dermatologist-diagnosed PsO were included at the outpatient and inpatient clinics of departments of dermatology of three University Medical Centers in Germany (Bonn, Frankfurt/Main, and Göttingen). The study sites were chosen as they were deemed representative of university medical centers in Germany with a sufficient number of psoriatic patients to include in the study. Moreover, the study sites were chosen as part of a network of collaborators who have successfully carried out clinical studies in the context of psoriasis in the past. This approach seemed to be most feasible considering limited resources for the study. Also, for comparison, a smaller cohort of patients with AD was recruited. Inclusion criteria comprised dermatologist-diagnosed PsO or AD, age ≥ 18 years, written informed consent, and the ability to fill out the questionnaire. Exclusion criteria comprised absence of diagnosis of PsO or AD, and underaged patients. Patients were enrolled consecutively and competitively across the study sites. The study was performed according to the principles of the Declaration of Helsinki [17] and approved by the Ethics Committee of all participating sites (Göttingen 40/2/19).

### 2.2. Data Collection

Data were collected from 08/2019 to 03/2020 via a paper-based questionnaire with respect to sociodemographic characteristics (age, gender and partnership status), socioeconomic characteristics (occupational status), disease characteristics (family history of PsO/AD, disease duration, disease severity (Psoriasis Area and Severity Index (PASI), Eczema Area and Severity Index (EASI)), health-related quality of life (Dermatology Life Quality Index (DLQI)), current and former dermatological therapy, and comorbidity (PsA, atopic, metabolic, psychiatric, and neoplastic diseases, and smoking status). Additionally, the participants indicated in the questionnaire whether they had previously suffered from infectious diseases (pneumonia, bronchitis, and herpes zoster; Supplementary File S1). Information with regard to disease characteristics, treatment history, and comorbidity was complemented by reviewing medical records. Moreover, patients were asked about their influenza vaccination status and their reasons for or against receiving an influenza vaccination. Information on vaccination status was additionally complemented by information from vaccination certificates, if available. As the yearly influenza vaccination may not have been documented in the vaccination certificates of some patients, discrepancies between questionnaire and vaccination certificates were solved by considering all patients with documentation of vaccination in the certificate and/or reported vaccination in the questionnaire as vaccinated.

### 2.3. Statistical Analysis

Pre-study statistical sample size calculation was not carried out formally since we aimed at performing a descriptive analysis. However, we wanted to include approximately *n* = 300 patients with PsO, assuming a vaccination rate of one-third in the sample, which enables meaningful subgroup analysis. This sample size is comparable to previous research in the field [16,18,19]. Statistical analysis was performed with opensource software RStudio^®^ (version 3.4.4, Boston, MA, USA) and figures were created with Prism^®^ (version 9.1.0, GraphPad Software, San Diego, CA, USA). Cohort and vaccination characteristics were analyzed descriptively. We did not apply any statistical methods of data imputation with regard to missing values. Due to checking questionnaires immediately after submission, missing values occurred very rarely and no participant had to be excluded due to missing data. Subgroup analyses were conducted with Pearson’s *χ*^2^-test for discrete-valued variables and Student’s *t*-test and Mann–Whitney U test for normally and not-normally distributed continuous variables, respectively. Regression analysis was performed to estimate the effect of the variables age, gender, occupational status, PsA, at least one further comorbidity (i.e., cardiovascular disease, asthma, COPD, hepatic cirrhosis, diabetes mellitus, chronic kidney disease, obesity, and neoplastic disease), a history of pneumonia, a history of bronchitis, and immunosuppressive therapy on the probability of an influenza vaccination. Moreover, we performed regression analysis to detect possible predictors (age, gender, occupational status, and PsA) of the most common reasons against influenza vaccination. Significance was assumed at *p* < 0.05.

## 3. Results

The overall participation rate was approximately 90%. Overall, 327 patients with PsO and 98 patients with AD were included (Table 1). The majority of patients were male (58.1% and 57.1% with PsO and AD, respectively). The average age amounted to 53.4 and 44.3 years, and the mean disease duration was 24.5 and 26.8 years for PsO and AD, respectively. Most PsO patients worked full-time (42.2%) or were retired (31.5%), while AD patients commonly worked full-time (36.7%), part-time (19.4%), were retired (18.4%), or were students (15.3%). With respect to comorbidity, PsA was diagnosed in more than half of all patients with PsO (57.2%). Comorbid metabolic and psychiatric diseases were frequently reported, particularly in patients with PsO, while atopic diseases (rhinoconjunctivitis, food allergies, and asthma) were frequently observed in patients with AD (Table 1). A total of 17.1% and 18.4% of all patients with PsO and AD indicated that they had suffered from pneumonia, respectively, while a history of bronchitis was reported by 28.7% and 29.6%, respectively. Herpes zoster occurred in 17.1% and 13.3% of patients with PsO and AD, respectively.

Disease activity was well-controlled and disease-related quality of life was high in PsO with a median PASI of 1.8 and a median DLQI of 3.0, while patients with AD had a median EASI of 10.6, indicating moderate disease, and a median DLQI of 12.0 (Table 2). Almost all PsO patients received systemic therapy at time of data collection (92.0%), particularly biologics (74.0%). Among biologics, interleukin (IL)-12/23 or IL23p19 antagonists were administered most frequently (34.9%), followed by tumor necrosis factor (TNF)-α antagonists (19.9%), and IL-17(R) inhibitors (19.0%). AD was less commonly treated systemically (39.8%), particularly with the IL-4/13 receptor inhibitor dupilumab (29.6%) and cyclosporine (8.2%). Treatment experience was high in both PsO and AD patients (Table 2).

Indication for influenza vaccination was present as standard indication by age (≥60 years) in 110 of all 327 PsO patients and 20 of all 98 AD patients, respectively, and in the younger patients due to immunosuppressive therapy, comorbidity, occupation, and/or pregnancy in 206 of 217 PsO patients (94.9%) and 56 of 78 AD patients (71.8%), respectively.

Approximately two-thirds of patients could present their vaccination certificates (69.7% and 70.4% for PsO and AD patients, respectively), while 26.9% and 19.4% of PsO and AD patients reported that they did not have or could not find their vaccination certificates.

**Figure 1:**
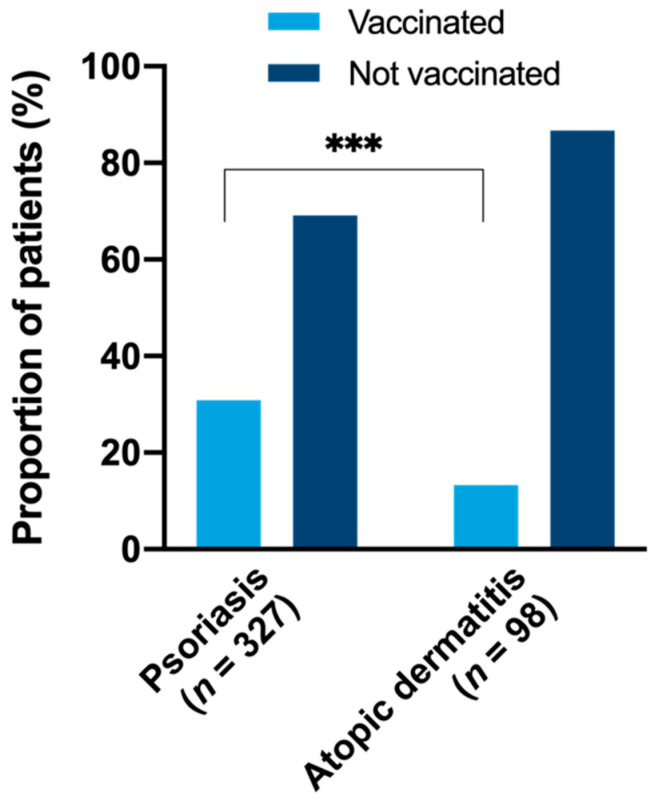
Influenza vaccination rate in season 2018/2019. Patients with an influenza vaccination in season 2018/2019 according to the questionnaire and/or vaccination certificate were categorized as vaccinated. The vaccination prevalence was significantly higher in PsO patients compared to AD patients (30.9% vs. 13.3%). *** *p* < 0.001).

**Figure 2:**
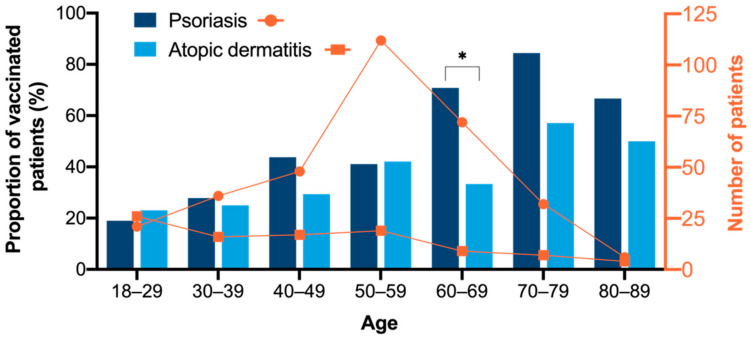
Influenza vaccination rate according to age groups. Patients with an influenza vaccination at some point according to the questionnaire and/or vaccination certificate were categorized as vaccinated. The bars depict the proportion of vaccinated patients in various age groups, and the curves show the total number patients in the respective age group. The proportion of vaccinated patients increased with rising age in the psoriasis patient group as well as in the atopic dermatitis patient group. In the age group 60–69 years, the proportion of vaccinated patients was significantly higher in PsO compared to AD patients (70.8% vs. 33.3%, *p* = 0.024). * *p* < 0.05.

Overall, 49.8% of patients with PsO (163/327) and 32.7% of patients with AD (32/98, *p* = 0.003) had received an influenza vaccination at some point according to the questionnaire and/or vaccination certificate. A total of 30.9% of patients with PsO (101/327) and 13.3% of patients with AD (13/98) were vaccinated in the previous season (2018/2019; *p* < 0.001) (Figure 1). Subgroup analysis with regard to patient and disease characteristics is provided in Supplementary Table S2. The probability of vaccination depended significantly on the age of participants with a higher mean age in vaccinated subgroups (mean age (SD) PsO: vaccinated: 58.0 years (12.5) vs. non-vaccinated: 48.8 years (13.6), *p* < 0.001; AD: vaccinated: 50.0 years (19.2) vs. non-vaccinated: 41.6 years (17.6), *p* = 0.040). The proportion of patients with influenza vaccination increased from 19.0% in the age group 18–29 years to 84.4% in the age group 70–79 years for PsO with a similar trend in AD (Figure 2). Overall, the vaccination prevalence was 74.5% in patients ≥60 years compared to 37.3% in patients < 60 years in PsO, *p* < 0.001 (in AD: 45.0% for ≥60 years and 29.5% for < 60 years, *p* = 0.187). The probability of influenza vaccination was by tendency higher in females than males. Vaccinated PsO patients were more likely to be retired than unvaccinated PsO patients and less likely to work full-time. Parameters of disease activity and quality of life were not significantly different between vaccinated and unvaccinated patients. In univariate analysis, compared to non-vaccinated patients, vaccinated individuals with PsO suffered significantly more frequently from PsA, obesity, arterial hypertension, dyslipidemia, diabetes mellitus, and COPD (Table S2). Comorbidity was not significantly associated with influenza vaccination status in AD. Among infectious diseases, previous bronchitis was more often reported by vaccinated subjects with PsO than by unvaccinated individuals (Table S2).

In multivariate regression analysis for patients with PsO, only higher age (*p* < 0.001) and a history of bronchitis (*p* = 0.023) were significantly associated with a higher probability of an influenza vaccination (Table 3). Gender, PsA, further comorbidity (i.e., at least one of the following: cardiovascular disease, asthma, COPD, hepatic cirrhosis, diabetes mellitus, chronic kidney disease, obesity, and neoplastic disease), a history of pneumonia, and immunosuppressive therapy did not predict influenza vaccination significantly.

**Figure 3:**
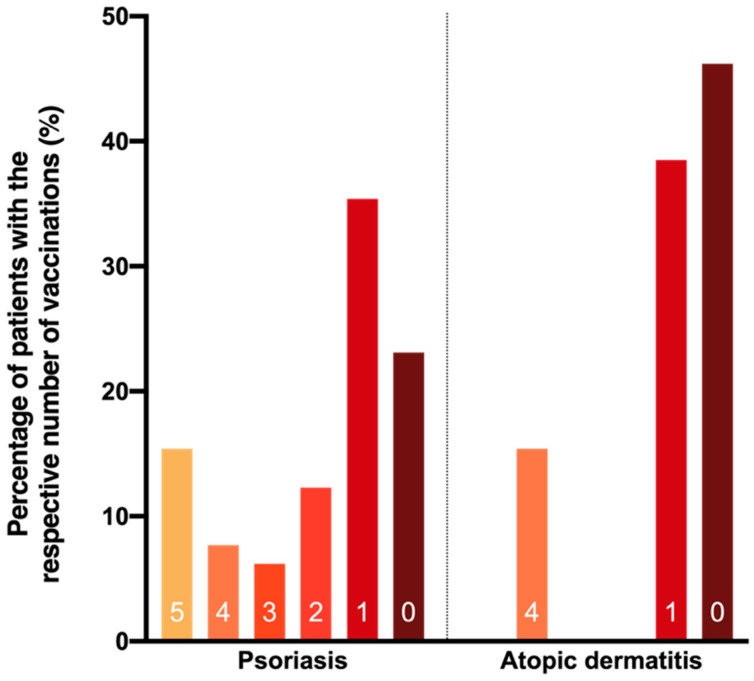
Number of influenza vaccinations per patient during the past five years according to vaccination certificates. The graph shows how many patients have received one, two, three, four, or five individual vaccinations in total within the past 5 years. The brown bars show how many patients have not been vaccinated within the past five years but have received at least one vaccination before that. Only influenza vaccinations according to the vaccination certificate and not the questionnaire were counted. A total of 163 (71.5%) psoriasis patients and 56 (81.2%) atopic dermatitis patients have never been vaccinated in the past, according to vaccination certificates.

According to vaccination certificates, 28.5% of patients with PsO and 18.8% of patients with AD had received an influenza vaccination at least once, and 21.9% and 10.1% were vaccinated within the past five years. Figure 3 depicts the number of patients who received one to five influenza vaccinations within in the past five years as well as the number of patients who received an influenza vaccination at some point but not within the past five years. A total of 10.1% of patients with PsO had one and 11.9% at least two vaccinations within the past five years (for AD: 7.2% and 2.9%, respectively). In addition, 7.6% of influenza vaccinations were received by PsO patients treated with non-biological systemic immunosuppressive medication at that time (i.e., cyclosporine, fumaric acid ester, leflunomide, methotrexate, tofacitinib), and 42.4% were received by PsO patients treated with biologics, respectively. However, in 12.5% of patients, it could not be determined whether vaccination was performed under systemic immunosuppressive therapy due to incomplete documentation.

**Figure 4:**
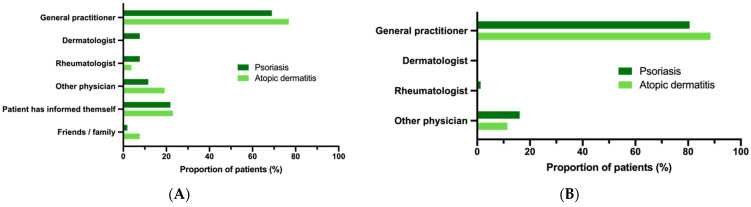
Recommendation and execution of influenza vaccination: (**A**) The total number of patients according to recommendation of an influenza vaccination by a health care provider or friends/family is depicted. Multiple indications were permitted; (**B**) The graph shows the health care provider who performed the influenza vaccination.

**Figure 5:**
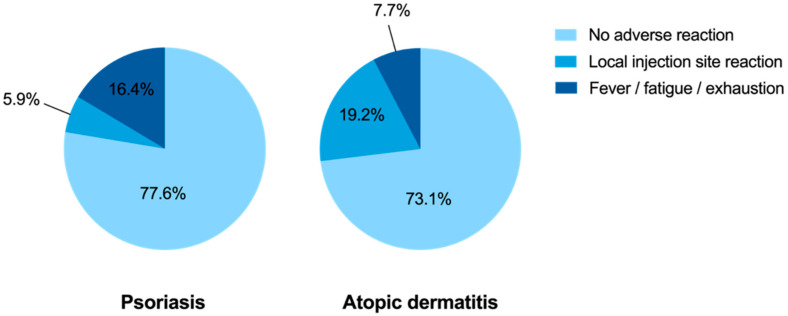
Tolerability of influenza vaccination. The pie charts show the tolerability of influenza vaccination in psoriasis and atopic dermatitis patients. Approximately three-quarters of patients reported no adverse reaction. There were no serious health disorders.

The recommendation for influenza vaccination had been given by the general practitioner in the majority of patients (69.0% for PsO and 76.9% for AD; Figure 4a) and similarly, almost all vaccinations were performed by the general practitioner (80.6% for PsO and 88.5% for AD; Figure 4b). Approximately three-quarters of vaccinated individuals reported no adverse reactions. Local reactions at the injection site were reported by more patients with AD compared to those with PsO (19.2% vs. 5.8%, *p* = 0.016), and systemic symptoms such as fever, fatigue, and exhaustion were reported by tendency more often by patients with PsO compared to AD patients (16.1% vs. 7.7%, *p* = 0.250; Figure 5).

Reasons for and against influenza vaccinations did not differ between the PsO and AD subgroups (Table 4). The predominant reasons for influenza vaccinations were the general recommendation to be vaccinated (53.0%) and the physician’s advice in the individual case (39.8%), while few patients reported the skin disease or its therapy as reason for vaccination (5.0% and 8.3%, respectively). On the other hand, the most commonly mentioned reasons against an influenza vaccination were the perception that the vaccination was not necessary in the individual case (54.5%), a personal history lacking a severe flu (43.0%), lack of awareness raised by the physician (40.2%), lack of confidence in the vaccine’s protective effect (23.0%), and concerns regarding tolerability (22.1%). Regression analysis revealed that patients who indicated lacking a recommendation by a physician as reason against vaccination were significantly younger than those who did not state this reason (*p* = 0.038), and patients who stated “no time for vaccination” as reason against vaccination were more likely to be working full-time (*p* = 0.006, Table S3). Patients who had been vaccinated at some point in the past but not in the previous season stated as reasons against the vaccination in the previous season most frequently concerns regarding tolerability, oblivion, and lacking time (31.9%, 18.8%, and 27.5%, respectively). Among patients who received an influenza vaccination at some point and stated no adverse reactions, 73.7% received an influenza vaccination in the previous season 2018/2019 as well, compared to 32.4% who reported any adverse reaction such as local reactions at the injection site, fever, fatigue, or exhaustion (*p* < 0.001).

## 4. Discussion

The main finding of this study is the determination of the influenza vaccination rate in patients with PsO with a life-time prevalence of 49.8% and a prevalence in the previous season (2018/2019) of 30.9%. In comparison, Radtke and colleagues reported an influenza vaccination rate in psoriatic patients recruited through the German registry “PsoBest” of 28% for the season 2010/2011 [16]. The slightly higher rate in our cohort may be partly attributable to differences in the respective cohorts such as a higher rate of PsA and systemic treatment in our cohort, the different setting of recruitment, and different influenza seasons analyzed. Interestingly, the age of participants was comparable to our study (57.7 years in vaccinated and 49.5 years in non-vaccinated individuals [16] vs. 58.0 years and 48.8 years in our study, respectively).

The German Health Interview and Examination Survey for Adults 2008–2011 (DEGS1) examined vaccination coverage in the German general population [21]. The reported influenza vaccination lifetime prevalence was 44.7%, with a higher prevalence in the age groups 60–69 years (63.8%) and 70–79 years (68.3%). According to German health insurance claims data, the prevalence of influenza vaccination in individuals ≥ 60 years of age started to decline from approximately 50% in the season 2008/2009 to 38.8% in the season 2019/2020 [22]. In adults with chronic diseases, the seasonal influenza vaccination prevalence was approximately 30% over the same period [22], which is comparable to our study. These data differ substantially from the WHO target of a coverage of at least 75% for older persons and the chronically ill [23].

Influenza vaccination has been previously studied in other chronic inflammatory diseases such as rheumatoid arthritis. A recent review on 16 studies found influenza vaccination rates ranging from 25.3 to 93.0% (mean: 59.5%), which is considerably higher than in our cohort [15].

In accordance with subgroup analysis, our regression model showed that age ≥ 60 years was by far the most important variable to predict an influenza vaccination in our cohort. Similarly, Radtke and colleagues reported a higher age in vaccinated PsO patients [16]. Noe and colleagues investigated influenza vaccination in PsO patients compared to other chronic diseases according to U.S.-based claims data [24]. They identified age as a significant predictor of vaccination in regression analysis as well. In our study, working full-time was associated with a lower influenza vaccination probability in subgroup analysis but not in the regression model, which suggests that the impact of occupational status might be primarily mediated by the age of participants. Interestingly, further risk factors that are associated with a complicated course of influenza infection and thus are an indication for vaccination irrespective of the age according to the RKI (i.e., PsA, cardiovascular disease, asthma, COPD, hepatic cirrhosis, diabetes mellitus, chronic kidney disease, obesity, and neoplastic disease immunosuppressive therapy) did not predict influenza vaccination in our regression model, while a personal history of bronchitis was associated with vaccination. In contrast, Noe and colleagues reported a range of comorbidities (asthma, chronic liver disease, diabetes, cancer, human immunodeficiency virus, and PsA) as significant predictors of influenza vaccination [24]. Interestingly, probability of vaccination did not depend on systemic antipsoriatic treatment in their study as well [24].

Our data suggest that the age limit of 60 years beyond which influenza is a standard vaccination is well known by patients and/or their general physicians, leading to a higher influenza vaccination rate in this older patient group. On the other side, additional indications for influenza vaccination in younger patients were not significant predictors in our model when controlled for age. This is of particular importance considering that the vast majority of patients below the age of 60 years had at least one additional indication for influenza vaccination. Treatment of psoriatic disease and associated comorbidity are well known by dermatologists and rheumatologists as experts in managing this complex disorder. However, given that influenza vaccinations were most frequently recommended by general physicians or the social environment but infrequently by dermatologists or rheumatologists, and that vaccination was commonly carried out by general physicians, there appears to be a need for a higher awareness for indication vaccination and closer interaction between different health care providers and patients. The need for awareness of particularly indication for vaccination is also reflected in our study, as only few patients indicated the skin disease, its treatment, or comorbidity as reason for vaccination, while, on the other side, the majority of unvaccinated individuals, particularly younger patients, reported a lack of personal information or recommendation as reasons for lack of vaccination. Moreover, it should be considered that in younger PsO patients, the dermatologist may be the only healthcare provider they see regularly, suggesting that dermatologists may be an important resource to provide counseling about vaccination.

Influenza vaccination was well-tolerated in our cohort with local injection site reactions and systemic symptoms such as fever reported by approximately one-third of patients, while there were no serious health disorders. According to the literature, there seems to be a low risk of mild exacerbation of psoriatic disease following influenza vaccination, which should be kept in mind when counseling patients in order to improve acceptance of vaccination [25].

Several limitations have to be kept in mind when interpreting the results of this study. First, PsO patients were compared to AD patients, which differed substantially in sociodemographic characteristics, and not an age- and sex-matched control group of the general population as this was the most feasible method. Second, the limited number of patients and the setting of disproportional recruitment in three specialized dermatological centers have to be considered. Third, the study relied partially on self-reported information, which bears a risk of imprecision and recall bias. Fourth, the life-time prevalence of influenza vaccination is a function of age, which might lead to overestimation of the difference of life-time vaccination prevalence between young individuals and the elderly. Fifth, although we adjusted for a wide range of patient and treatment characteristics, possibly relevant associated variables may have been neglected (e.g., socioeconomic status). Finally, not all influenza vaccinations were documented in the vaccination certificate, and a substantial number of patients could not present any vaccination certificate.

A major strength is the multicenter, cross-sectional design of the study. The vaccination status was carefully evaluated using both patient reports and vaccination certificates with extensive subgroup analysis with regard to patient and disease characteristics.

In conclusion, our study adds to the growing body of literature that (1) influenza vaccination rates among patients with PsO remain inadequate and (2) that this applies particularly to individuals outside the standard influenza vaccination (i.e., younger than 60 years) but with indication vaccination recommendation (i.e., comorbidity, immunosuppressive therapy). Moreover, our data confirm (3) the pivotal role of the general physician in recommending and performing vaccinations and (4) the potential role of the dermatologist in raising awareness and providing counseling.

In a time of a global pandemic with several COVID-19 vaccines currently available, the significance of vaccination cannot be understated. Further studies on the general vaccination status and perceptions of psoriatic patients with a longitudinal design to compare the pre- and post-COVID-19 era would be desirable.

## Figures and Tables

**Table 1:** Cohort characteristics. ^a^ If not indicated otherwise, the number and percentage are depicted. ^b^ Overall, *n* = 347 patients were recruited in Göttingen, *n* = 58 in Bonn, and *n* = 20 in Frankfurt. ^c^ Cardiovascular diseases comprised heart attack, stroke, coronary artery disease, arterial occlusive disease, cardiac insufficiency. ^d^ Other hepatic diseases comprised focal nodular hyperplasia (*n* = 1), Gilbert’s syndrome (*n* = 1), hemangioma (*n* = 2), primary biliary cholangitis (*n* = 1), hepatitis B (*n* = 7), hepatitis C (*n* = 2), and healed hepatitis E (*n* = 2). ^e^ Chronic kidney disease was assumed if indicated in the questionnaire and, if not indicated in the questionnaire, according to serum creatinine levels using the CKD-EPI equation 2009 [20]. ^f^ Neoplastic diseases comprised lymphoma (*n* = 4), breast cancer (*n* = 4), cervix carcinoma (*n* = 2), melanoma (*n* = 2), renal cell carcinoma (*n* = 1), colon carcinoma (*n* = 1) and glioblastoma (*n* = 1). ^g^ The percentage refers to the number of patients with the respective infectious disease (pneumonia, bronchitis, and herpes zoster, respectively). PsO: psoriasis; AD: atopic dermatitis; PsA: psoriatic arthritis; BMI: Body mass index; COPD: chronic obstructive pulmonary disease; SD: standard deviation.

**Characteristic**	**Psoriasis, *n* (%) ^a^**	**Atopic Dermatitis, *n* (%) ^a^**
Cohort size ^b^	327	98
Female gender	137 (41.9)	42 (42.9)
Age, years, mean (SD)	53.4 (13.9)	44.3 (18.5)
Age at onset of disease, years, mean (SD)	28.9 (16.8)	17.5 (25.0)
Disease duration, years, mean (SD)	24.5 (15.5)	26.8 (18.0)
Family history of PsO	163 (49.8)	21 (21.4)
Family history of AD	50 (15.3)	46 (46.9)
Occupational status		
Working full-time	138 (42.2)	36 (36.7)
Working part-time	38 (11.6)	18 (18.4)
Currently unemployed	11 (3.4)	3 (3.1)
Student	14 (4.3)	15 (15.3)
Retired	103 (31.5)	18 (18.4)
Unable to work	10 (3.1)	4 (4.1)
Self-employed	13 (4.0)	4 (4.1)
Partnership status		
In a permanent relationship	245 (74.9)	73 (74.5)
Not in a permanent relationship	65 (19.9)	22 (22.4)
Widowed	17 (5.2)	3 (3.1)
Comorbidity		
PsA	187 (57.2)	-
Age at onset of PsA, years, mean (SD)	46.1 (13.6)	-
BMI, mean (SD)	29.9 (6.6)	26.0 (5.5)
Obesity (BMI ≥ 30)	142 (43.4)	15 (15.3)
Arterial hypertension	146 (44.6)	30 (30.6)
Cardiovascular disease ^c^	56 (17.1)	12 (12.2)
Dyslipidemia	86 (26.3)	11 (11.2)
Hepatic steatosis	106 (32.4)	7 (7.1)
Hepatic cirrhosis	7 (2.1)	0 (0.0)
Other hepatic diseases ^d^	14 (4.3)	2 (2.0)
Diabetes mellitus	40 (12.2)	4 (4.1)
Chronic kidney disease ^e^	20 (6.3)	4 (5.4)
COPD/emphysema	31 (9.5)	7 (7.1)
Asthma	29 (8.9)	41 (41.8)
Allergic contact dermatitis	28 (8.6)	38 (38.8)
Allergic rhinoconjunctivities	52 (15.9)	61 (62.2)
Food allergies	27 (8.3)	47 (48.0)
Depression	70 (21.4)	16 (16.3)
Neoplastic diseases ^f^	11 (3.4)	4 (4.1)
Smoking status		
Current smoker	112 (34.3)	31 (31.6)
Ex-smoker	144 (44.0)	27 (27.6)
Never smoker	71 (21.7)	40 (40.8)
Previous infectious diseases		
Pneumonia	56 (17.1)	18 (18.4)
Years since pneumonia, mean (SD)	15.7 (15.4)	14.8 (8.4)
Hospitalization due to pneumonia ^g^	14 (25.0)	7 (43.8)
Bronchitis	94 (28.7)	29 (29.6)
Years since bronchitis, mean (SD)	7.7 (9.8)	6.2 (8.8)
Hospitalization due to bronchitis ^g^	8 (8.7)	1 (3.6)
Herpes zoster	56 (17.1)	13 (13.3)

**Table 2:** Disease characteristics. ^a^ If not indicated otherwise, the number and percentage are depicted. ^b^ Other biologicals in the PsO group comprised abatacept (*n* = 1). ^c^ Other therapy in the PsO group comprised efalizumab (*n* = 12), onercept (*n* = 2), mycophenolatmofetil (*n* = 1), alefacept (*n* = 2), abatacept (*n* = 1), anakinra (*n* = 1), and rituximab (*n* = 1). Other Therapy in the AD group comprised autohaemotherapy (*n* = 1), nemolizumab (*n* = 1), baricitinib (*n* = 1), and azathioprin (*n* = 1). IQR: interquartile range; NA: not applicable.

Characteristic	Psoriasis, *n* (%) ^a^	Atopic Dermatitis, *n* (%) ^a^
Disease activity and quality of life		
PASI, median (IQR)	1.8 (0.2–4.0)	-
EASI, median (IQR)	-	10.6 (2.7–17.2)
DLQI, median (IQR)	3.0 (0.0–9.0)	12.0 (5.0–18.0)
Current therapy		
Phototherapy	1 (0.3)	9 (9.2)
Systemic therapy	301 (92.0)	39 (39.8)
Apremilast	15 (4.6)	-
Cyclosporine	0 (0.0)	8 (8.2)
Fumaric acid ester	17 (5.2)	-
Leflunomide	1 (0.3)	-
Methotrexate	17 (5.2)	1 (1.0)
Tofacitinib	6 (1.8)	-
Retinoids	3 (0.9)	1 (1.0)
Biologics ^b^	242 (74.0)	29 (29.6)
TNF-α antagonists	65 (19.9)	-
IL-(12)/23 antagonists	114 (34.9)	-
IL-17(R) antagonists	62 (19.0)	-
Dupilumab	-	29 (29.6)
Former therapy		
Phototherapy	152 (46.5)	48 (49.0)
Systemic therapy	287 (87.8)	42 (42.9)
Apremilast	31 (9.5)	-
Corticosteroids	NR	24 (24.5)
Cyclosporine	43 (13.1)	20 (20.4)
Fumaric acid ester	164 (50.2)	-
Leflunomide	15 (4.6)	-
Methotrexate	175 (53.5)	5 (5.1)
Sulfasalazine	12 (3.7)	-
Tofacitinib	1 (0.3)	-
Retinoids	50 (15.3)	5 (5.1)
Biologics	181 (55.4)	6 (6.1)
TNF-α antagonists	130 (39.8)	-
IL-12/23 antagonists	79 (24.2)	-
IL-17(R) antagonists	55 (16.8)	-
Dupilumab	-	6 (6.1)
Other therapy ^c^	20 (6.1)	6 (6.1)
Number of previous therapies, mean (SD)	3.7 (2.6)	3.6 (1.9)

**Table 3:** Regression analysis predicting influenza vaccination in PsO patients. The probability of an influenza vaccination was chosen as the dependent variable. Age, gender, occupational status, PsA, at least one further comorbidity (i.e., cardiovascular disease, asthma, COPD, hepatic cirrhosis, diabetes mellitus, chronic kidney disease, obesity, and neoplastic disease), a history of pneumonia, a history of bronchitis, and immunosuppressive therapy served as independent variables. Significant predictors are printed in bold. ^a^ The reference category to female was male. ^b^ The reference category of working full-time was any other occupational status. ^c^ Only current immunosuppressive or immunomodulatory systemic therapy administered for PsO or PsA was considered (i.e., cyclosporine, fumaric acid ester, leflunomide, methotrexate, tofacitinib, and biologicals). The model was adjusted for the center of recruitment (not significant). SE: standard error.

Predictor	Coefficient (SE)	*p*-Value
**Age ≥ 60 years**	**0.356 (0.063)**	**<0.001**
Female ^a^	0.050 (0.055)	0.366
Working full-time ^b^	0.010 (0.065)	0.875
PsA	0.084 (0.054)	0.120
Further comorbidity	0.016 (0.069)	0.820
History of pneumonia	0.027 (0.072)	0.712
**History of bronchitis**	**0.136 (0.059)**	**0.023**
Immunosuppressive therapy ^c^	0.016 (0.069)	0.820
Constant	0.109 (0.120)	0.362

**Table 4:** Reasons for and against influenza vaccination. ^a^ Other reasons in the psoriasis patient group comprised “required by employer” (*n* = 10), “offered by employer“ (*n* = 3), “frequent contact with people“ (*n* = 4), and “experience with influenza infection in other people or themselves” (*n* = 4). Other reasons in the atopic dermatitis patient group comprised “required by employer” (*n* = 6) and “immunosuppression of a family member” (*n* = 1). ^b^ Other reasons in the psoriasis patient group comprised “patient does not want to take too many pharmaceuticals” (*n* = 1) and “vaccine not available” (*n* = 1). Other reasons in the atopic dermatitis patient group comprised “allergy to vaccine” (*n* = 3), “pregnancy” (*n* = 1), and “vaccine not available” (*n* = 1).

Reasons for Vaccination	Psoriasis, *n* (%)	Atopic Dermatitis, *n* (%)
General recommendation	82 (52.9)	14 (53.8)
Physician’s advice	60 (38.7)	12 (46.2)
Skin disease	9 (5.8)	0 (0.0)
Treatment of skin disease	14 (9.0)	1 (3.8)
Comorbidity/comedication	19 (12.3)	4 (15.4)
Other reasons ^a^	21 (135)	7 (26.9)
Reasons against vaccination		
Vaccination not deemed necessary by patient	96 (55.8)	37 (51.4)
No personal history of severe flu	74 (43.0)	31 (43.1)
Lacking recommendation by a physician	69 (40.1)	29 (40.3)
Lacking confidence in protective effect	41 (23.8)	15 (20.8)
Potential side effects	41 (23.8)	13 (18.1)
Patient forgot to get vaccinated	18 (10.5)	9 (12.5)
No time for vaccination	18 (10.5)	9 (12.5)
Advised not to get vaccinated by a physician	9 (5.2)	7 (9.7)
(Co)payment	4 (2.3)	0 (0.0)
Inflammatory activity of skin disease	4 (2.3)	4 (5.6)
Treatment of skin disease	12 (7.0)	1 (1.4)
Comorbidity/comedication	10 (5.8)	7 (9.8)
Other reasons ^b^	2 (1.2)	5 (6.9)

## Data Availability

Not applicable.

## Supplementary Materials

The following are available online at https://www.mdpi.com/article/10.3390/vaccines9080843/s1, File S1. Patient questionnaire (German version). Table S2. Subgroup analysis with respect to influenza vaccination status. Table S3. Regression analyses predicting reasons against influenza vaccination in PsO patients.
