# Supplementary material for: Influenza Vaccination in Psoriatic Patients—Epidemiology and Patient Perceptions: A German Multicenter Study (Vac-Pso)
Source: Vaccines (Basel). 2021 Aug 1;9(8):843. doi: 10.3390/vaccines9080843 (PMC8402561; doi:10.3390/vaccines9080843)
Supplement: Supplementary file 1 [file vaccines-09-00843-s001.zip › vaccines-1294367-supplementary.pdf]

vaccines MDPI

Supplementary Material

File S1. Patient questionnaire (German version)

Zentrum: ___________

Patienten-ID: ________

UNIVERSITÄTSMEDIZIN GÖTTINGEN UMG
Klinik für Dermatologie, Venerologie und Allergologie

Patientenfragebogen

Für die Studie:

Pneumokokkenimpfung bei Patienten mit Psoriasis oder atopischer Dermatitis

1. **Welches ist Ihr Geschlecht?**
   - männlich
   - weiblich

2. **Bitte nennen Sie Ihren Geburtsmonat** (z.B. 01 für Januar) **und Ihr Geburtsjahr** (z.B. 1950)**.**

   Geburtsmonat: ___________________

   Geburtsjahr: _____________________

3. **Wie alt waren Sie bei Beginn der ersten Symptome Ihrer Hauterkrankung?**
   ________ Jahre

4. **Ist in Ihrer Familie eine Person von Psoriasis („Schuppenflechte") betroffen?**
   - Nein
   - Ja

5. **Ist in Ihrer Familie eine Person von atopischer Dermatitis („Neurodermitis") betroffen?**
   - Nein
   - Ja

6. **Wurde bei Ihnen eine Gelenkbeteiligung bei Psoriasis (psoriatisches Gelenkrheuma, „Psoriasisarthritis") durch einen Arzt festgestellt?**
   - Nein
   - Ja, die Diagnose wurde im Alter von ________________ Jahren festgestellt

7. **Wie ist Ihr derzeitiger Familienstand?**
   - Feste Partnerschaft / verheiratet
   - Derzeit in keiner festen Beziehung (inkl. geschieden und dauerhaft getrennt) lebend
   - Verwitwet

Zentrum: ___________

Patienten-ID: ________

UNIVERSITÄTSMEDIZIN GÖTTINGEN UMG
Klinik für Dermatologie, Venerologie und Allergologie

8. Welchen höchsten allgemeinbildenden Schulabschluss haben Sie?
- o Keinen Schulabschluss
- o Hauptschulabschluss
- o Realschulabschluss (Mittlere Reife)
- o Fachhochschulreife
- o Abitur/Allgemeine oder fachgebundene Hochschulreife
- o Anderer Schulabschluss. Wenn ja, welchen: _______________

9. Welchen beruflichen Ausbildungsabschluss haben Sie?
- o Noch in beruflicher Ausbildung (Auszubildende/r, Student/in)
- o Abgeschlossene Lehre oder Ausbildung
- o Meister-, Techniker-, oder gleichwertigen Fachschulabschluss
- o Hochschulabschluss
- o Fachhochschulabschluss (auch Abschluss einer Ingenieurschule)
- o keine abgeschlossene Berufsausbildung
- o Anderen beruflichen Abschluss. Wenn ja, welchen: ______________

10. Welcher der folgenden Kategorien würden Sie sich am ehesten zuordnen?
- o Vollzeit erwerbstätig
- o Teilzeit erwerbstätig
- o Nicht erwerbstätig
- o Student / Auszubildender
- o Rentner
- o Arbeitsunfähig
- o Selbständig

11. Wie sind Sie krankenversichert?
- o Gesetzlich
- o Privat
- o Gesetzlich mit privater Zusatzversicherung

12. Welche anderen Erkrankungen sind bei Ihnen bekannt (Mehrfachnennungen möglich)**?**

Ja	Erkrankung
O	Allergisches Kontaktekzem
O	Allergische Rhinokonjunktivitis („Heuschnupfen“)
O	Lebensmittelallergien
O	Erhöhter Blutdruck
O	Herz-Kreislauferkrankungen (Herzinfarkt, Schlaganfall, koronare Herzerkrankung, arterielle Verschlusskrankheit, Herzschwäche)
O	Erhöhte Cholesterinwerte / Blutfettwerte
O	Depressionen
O	Asthma
O	Chronische Bronchitis (COPD) oder Lungenemphysem
O	Fettleber
O	Leberzirrhose

Zentrum: ___________

Patienten-ID: ________

UNIVERSITÄTSMEDIZIN GÖTTINGEN UMG
Klinik für Dermatologie, Venerologie und Allergologie

Ja	Erkrankung
O	andere Lebererkrankung
	wenn ja, welche: ___________
O	Diabetes mellitus
O	Falls ja, müssen Sie Insulin spritzen?
O	Chronisches Nierenversagen
O	Windpocken
O	Krebs
	wenn ja, welcher: ___________

13. Wie groß sind Sie?
_____________cm

14. Welches Gewicht haben Sie?
_____________kg

15. Hatten Sie jemals eine Lungenentzündung („Pneumonie"), die mit Antibiotika behandelt werden musste (hierzu zählen keine leichten grippalen Infekte)**?**
- Ich weiß nicht
- Nein
- Ja, vor etwa _______________Jahren
  Falls ja, wurden Sie deswegen stationär in einem Krankenhaus behandelt?
  - Nein, nur ambulant.
  - Ja, stationär.

16. Hatten Sie jemals eine schwere Entzündung der Atemwege („schwere Bronchitis"), die mit Antibiotika behandelt werden musste (hierzu zählen keine leichten grippalen Infekte)**?**
- Ich weiß nicht
- Nein
- Ja, vor etwa _______________Jahren
  Falls ja, wurden Sie deswegen stationär in einem Krankenhaus behandelt?
  - Nein, nur ambulant.
  - Ja, stationär.

17. Hatten Sie jemals eine Gürtelrose („Herpes Zoster")?
- Ich weiß nicht
- Nein
- Ja, vor etwa _______________Jahren
  Falls ja, war die Gürtelrose bei Ihnen mit Schmerzen verbunden („Zosterneuralgie")?
  - Nein
  - Ja

18. Wissen Sie, ob Sie einen positiven Bluttiter bzgl. Varizellen (Windpocken) haben (Dieser wird z.B. im Rahmen einer Schwangerschaft bestimmt)**?**
- Ja, ich habe einen positiven Titer.
- Ja, ich habe einen negativen Titer.
- Ich weiß nicht.

Zentrum: ___________

Patienten-ID: ________

UNIVERSITÄTSMEDIZIN GÖTTINGEN UMG
Klinik für Dermatologie, Venerologie und Allergologie

19. Rauchen Sie zurzeit?

- o Ja, 1 - 10 Zigaretten pro Tag
- o Ja, 11 - 20 Zigaretten pro Tag
- o Ja, über 20 Zigaretten pro Tag
- o Ja, E-Zigaretten
- o Nein, aber ich habe früher geraucht
- o Nein, ich habe noch nie geraucht

20. Sind Sie gegen Influenza geimpft (dies entspricht der „Grippeschutzimpfung“)?

- o Ja, und Impfungen wurden in den Impfausweis eingetragen → Bitte folgen Sie dem Pfeil links.
- o Ja, aber Impfungen wurden nicht in den Impfausweis eingetragen → Bitte folgen Sie dem Pfeil links.
- o Nein → Bitte überspringen Sie die folgenden Fragen und machen mit Frage 27 (blau markiert) weiter (dem Pfeil rechts folgen).
- o Weiß ich nicht → Bitte überspringen Sie die folgenden Fragen und machen mit Frage 27 (blau markiert) weiter (dem Pfeil rechts folgen).

21. Wann wurde die Grippeschutzimpfung bei Ihnen durchgeführt?

Zuletzt vor _____________ Jahren

- o Weiß ich nicht

22. Wer hat Ihnen die Grippeschutzimpfung empfohlen? (Mehrfachnennungen möglich)

Ja	Nein	
O	O	Mein Hausarzt / Internist
O	O	Mein Dermatologe
O	O	Mein Rheumatologe
O	O	Ein anderer Arzt
O	O	Kein Arzt, ich habe mich selbst informiert
O	O	Bekannte

23. Wer hat die Grippeschutzimpfung bei Ihnen durchgeführt?

- o Mein Hausarzt / Internist
- o Mein Dermatologe
- o Mein Rheumatologe
- o Ein anderer Arzt

24. Aus welchem Grund haben Sie sich für die Grippeschutzimpfung entschieden (Mehrfachantworten möglich)**?**

Ja	Nein	
O	O	Aufgrund der allgemeinen Empfehlung zu dieser Impfung
O	O	Aufgrund der allgemeinen Empfehlung meines Arztes
O	O	Aufgrund meiner Hauterkrankung
O	O	Aufgrund der Behandlung meiner Hauterkrankung
O	O	Aufgrund anderer bei mir bekannter Erkrankungen oder deren Behandlung
O	O	Anderer Grund: ___________________

Zentrum: ___________

Patienten-ID: ________

UNIVERSITÄTSMEDIZIN GÖTTINGEN UMG
Klinik für Dermatologie, Venerologie und Allergologie

25. Wie haben Sie die Grippeschutzimpfung vertragen?

- o Die Impfung habe ich ohne Probleme vertragen
- o Nach der Impfung traten Reaktionen an der Einstichstelle auf (z.B. Rötung, Verhärtung, Schwellung)
- o Nach der Impfung traten Fieber, Abgeschlagenheit, Müdigkeit, Muskelschmerzen oder Kopfschmerzen auf
- o Nach der Impfung kam es zu einer schweren Störung des Gesundheitszustandes, nämlich: ____________________

26. Für den Fall, dass bei Ihnen im Winterhalbjahr 2018/2019 KEINE Grippeschutzimpfung durchgeführt wurde. Was war der Grund hierfür (Mehrfachnennungen möglich)?

- o Trifft nicht zu. Bei mir wurde im Winterhalbjahr 2018/2019 eine Grippeschutzimpfung durchgeführt.

ODER:

Trifft zu	Trifft nicht zu	
O	O	Mir wurde diese Impfung bisher nicht von einem Arzt empfohlen
O	O	Mir wurde von einem Arzt von der Impfung abgeraten
O	O	Ich halte diese Impfung für nicht notwendig.
O	O	Ich habe diese Impfung vergessen.
O	O	Ich hatte keine Zeit.
O	O	Ich hätte die Impfung selbst bezahlen müssen
O	O	Weil ich nicht glaube, dass mich die Impfung ausreichend vor der Infektion schützt
O	O	Aus Angst vor möglichen Nebenwirkungen
O	O	Aufgrund meines Hautzustandes war keine Impfung möglich
O	O	Aufgrund von Medikamenten, die ich zur Behandlung der Hauterkrankung regelmäßig einnehme
O	O	Aufgrund von anderen Medikamenten, die ich regelmäßig einnehme
O	O	Aufgrund von anderen Erkrankungen
O	O	Ich hatte bisher keine schwere Grippe
O	O	Anderer Grund: ______________________

27. Bei Ihnen wurde KEINE Grippeschutzimpfung durchgeführt. Bitte nennen Sie den Grund hierfür (Mehrfachnennungen möglich)**!**

Trifft zu	Trifft nicht zu	
O	O	Mir wurde diese Impfung bisher nicht von einem Arzt empfohlen
O	O	Mir wurde von einem Arzt von der Impfung abgeraten
O	O	Ich halte diese Impfung für nicht notwendig.
O	O	Ich habe diese Impfung vergessen.
O	O	Ich hatte keine Zeit.

Zentrum: ____________

Patienten-ID: _________

UNIVERSITÄTSMEDIZIN GÖTTINGEN UMG
Klinik für Dermatologie, Venerologie und Allergologie

Trifft zu	Trifft nicht zu	
O	O	Ich hätte die Impfung selbst bezahlen müssen
O	O	Weil ich nicht glaube, dass mich die Impfung ausreichend vor der Infektion schützt
O	O	Aus Angst vor möglichen Nebenwirkungen
O	O	Aufgrund meines Hautzustandes war keine Impfung möglich
O	O	Aufgrund von Medikamenten, die ich zur Behandlung der Hauterkrankung regelmäßig einnehme
O	O	Aufgrund von anderen Medikamenten, die ich regelmäßig einnehme
O	O	Aufgrund von anderen Erkrankungen
O	O	Ich hatte bisher keine schwere Grippe
O	O	Anderer Grund: __________________________

28. Sind Sie gegen Pneumokokken geimpft? (Pneumokokken sind bakterielle Erreger einer Lungenentzündung, dies entspricht NICHT der jährlichen „Grippeschutzimpfung")
- Ja → Bitte folgen Sie dem Pfeil links.
- Nein → Bitte überspringen Sie die folgenden Fragen und machen mit der letzten Frage weiter (rot markierte Frage 34, dem Pfeil rechts folgen)
- Weiß ich nicht → Bitte überspringen Sie die folgenden Fragen und machen mit der letzten Frage weiter (rot markierte Frage 34, dem Pfeil rechts folgen)

29. Wann wurde die Pneumokokkenimpfung bei Ihnen durchgeführt?

Zuletzt vor ________________ Jahren
- Weiß ich nicht

30. Wer hat Ihnen die Pneumokokkenimpfung empfohlen? (Mehrfachnennungen möglich)

Ja	Nein	
O	O	Mein Hausarzt / Internist
O	O	Mein Dermatologe
O	O	Mein Rheumatologe
O	O	Ein anderer Arzt
O	O	Kein Arzt, ich habe mich selbst informiert
O	O	Bekannte

31. Wer hat die Pneumokokkenimpfung bei Ihnen durchgeführt?
- Mein Hausarzt / Internist
- Mein Dermatologe
- Mein Rheumatologe
- Ein anderer Arzt

Zentrum: ___________

Patienten-ID: ________

UNIVERSITÄTSMEDIZIN GÖTTINGEN UMG
Klinik für Dermatologie, Venerologie und Allergologie

32. Aus welchem Grund haben Sie sich für die Pneumokokkenimpfung entschieden (Mehrfachantworten möglich)?

Ja	Nein	
O	O	Aufgrund der allgemeinen Empfehlung zu dieser Impfung
O	O	Aufgrund der allgemeinen Empfehlung meines Arztes
O	O	Aufgrund meiner Hauterkrankung
O	O	Aufgrund der Behandlung meiner Hauterkrankung
O	O	Aufgrund anderer bei mir bekannter Erkrankungen oder deren Behandlung
O	O	Anderer Grund: ___________________

33. Wie haben Sie die Pneumokokkenimpfung vertragen?

- o Die Impfung habe ich ohne Probleme vertragen
- o Nach der Impfung traten Reaktionen an der Einstichstelle auf (z.B. Rötung, Verhärtung, Schwellung)
- o Nach der Impfung traten Fieber, Abgeschlagenheit, Müdigkeit, Muskelschmerzen oder Kopfschmerzen auf
- o Nach der Impfung kam es zu einer schweren Störung des Gesundheitszustandes, nämlich: ____________________

34. Bei Ihnen wurde KEINE Pneumokokkenimpfung durchgeführt. Bitte nennen Sie den Grund hierfür (Mehrfachnennungen möglich)**!**

Trifft zu	Trifft nicht zu	
O	O	Mir wurde diese Impfung bisher nicht von einem Arzt empfohlen
O	O	Mir wurde von einem Arzt von der Impfung abgeraten
O	O	Ich halte diese Impfung für nicht notwendig.
O	O	Ich habe diese Impfung vergessen.
O	O	Ich hatte keine Zeit.
O	O	Ich hätte die Impfung selbst bezahlen müssen
O	O	Weil ich nicht glaube, dass mich die Impfung ausreichend vor der Infektion schützt
O	O	Aus Angst vor möglichen Nebenwirkungen
O	O	Aufgrund meines Hautzustandes war keine Impfung möglich
O	O	Aufgrund von Medikamenten, die ich zur Behandlung der Hauterkrankung regelmäßig einnehme
O	O	Aufgrund von anderen Medikamenten, die ich regelmäßig einnehme
O	O	Aufgrund von anderen Erkrankungen
O	O	Ich hatte bisher keine schwere Grippe
O	O	Anderer Grund: ______________________

Vielen Dank für Ihre Teilnahme an der Studie.

Vom Arzt auszufüllen: DLQI:__________________ PASI:______________________

Table S2. Subgroup analysis with respect to influenza vaccination status. [a] If not indicated otherwise, the number and percentage are depicted. [b] Differences comparing the subgroups of vaccinated and not vaccinated patients separately for PsO and AD were calculated with Pearson's χ^2-test for discrete-valued variables and student's t-test and Mann-Whitney u test for normally and not-normally distributed continuous variables, respectively. Significant results are printed in bold. [c] Cardiovascular diseases comprised heart attack, stroke, coronary artery disease, arterial occlusive disease, cardiac insufficiency.[d] Other hepatic diseases comprised focal nodular hyperplasia (n=1), Gilbert's syndrome (n=1), hemangioma (n=2), primary biliary cholangitis (n=1), hepatitis B (n=7), hepatitis C (n=2), and healed hepatitis E (n=2). [e] Chronic kidney disease was assumed if indicated in the questionnaire and, if not indicated in the questionnaire, according to serum creatinine levels using the CKD-EPI equation 2009 [23]. [f] Neoplastic diseases comprised lymphoma (n=4), breast cancer (n=4), cervix carcinoma (n=2), melanoma (n=2), renal cell carcinoma (n=1), colon carcinoma (n=1) and glioblastoma (n=1). [g] The percentage refers to the number of patients with the respective infectious disease (pneumonia, bronchitis, and herpes zoster, respectively). [h] Immunosuppressive therapy consisted of cyclosporine, fumaric acid ester, leflunomide, methotrexate, tofacitinib, and biologicals. PsO: psoriasis; AD: atopic dermatitis; PsA: psoriatic arthritis; BMI: Body mass index; COPD: chronic obstructive pulmonary disease; SD: standard deviation; IQR: interquartile range.

**Characteristic**	**Psoriasis**			**Atopic dermatitis**		
	**Vaccinated, n (%)[a]**	**Unvaccinated, n (%)[a]**	**p-value [b]**	**Vaccinated, n (%)[a]**	**Unvaccinated, n (%)[a]**	**p-value**
Cohort size	163	164		32	66	
Female gender	77 (47.2)	60 (36.6)	0.057	16 (50.0)	26 (39.4)	0.386
Age, years, mean (SD)	**58.0 (12.5)**	**48.8 (13.6)**	**<0.001**	**50.0 (19.2)**	**41.6 (17.6)**	**0.040**
Age at onset of disease, years, mean (SD)	**33.4 (17.7)**	**24.3 (14.5)**	**0.006**	21.9 (29.5)	15.4 (22.5)	0.271
Disease duration years, mean (SD)	24.6 (16.1)	24.4 (14.9)	0.922	28.1 (21.3)	26.2 (16.4)	0.664
Family history of PsO	75 (46.0)	88 (53.7)	0.185	6 (18.8)	15 (22.7)	0.795
Family history of AD	27 (16.6)	23 (14.0)	0.543	13 (40.6)	33 (50.0)	0.399
Occupational status						
Working full-time	**54 (33.1)**	**84 (51.2)**	**0.001**	10 (31.3)	26 (39.4)	0.507
Working part-time	15 (9.2)	23 (14.0)	0.227	7 (21.9)	11 (16.7)	0.583
Currently unemployed	7 (4.3)	4 (2.4)	0.379	2 (6.3)	1 (1.5)	0.248
Student	**2 (1.2)**	**12 (7.3)**	**0.011**	4 (12.5)	11 (16.7)	0.768
Retired	**73 (44.8)**	**30 (18.3)**	**0.002**	7 (21.9)	11 (16.7)	0.583
Unable to work	6 (3.7)	4 (2.4)	0.542	1 (3.1)	3 (45.5)	1.000
Self employed	6 (3.7)	7 (4.3)	1.000	1 (3.1)	3 (45.5)	1.000
Partnership status						
In a permanent relationship	123 (75.5)	122 (74.4)	0.899	**24 (75.0)**	**16 (24.2)**	**0.006**
no partner	28 (17.2)	37 (22.6)	0.268	**6 (18.8)**	**49 (74.2)**	**0.002**
widowed	12 (7.4)	5 (3.0)	0.087	2 (6.3)	1 (1.5)	0.248
**Comorbidity**						
PsA	**104 (63.8)**	**83 (50.6)**	**0.019**	-	-	-
Age at onset of PsA, years, mean (SD)	**49.3 (13.8)**	**42.2 (12.4)**	**<0.001**	-	-	-

BMI (mean, SD)	30.2 (6.6)	29.7 (6.6)	0.504	27.9 (7.6)	25.1 (3.9)	0.056
Obesity (BMI ≥ 30)	**82 (50.3)**	**60 (36.6)**	**0.014**	6 (18.8)	9 (13.6)	0.556
Arterial hypertension	**90 (55.2)**	**56 (34.1)**	**<0.001**	12 (37.5)	18 (27.3)	0.361
Cardiovascular disease[c]	32 (19.6)	24 (14.6)	0.244	5 (15.6)	7 (10.6)	0.520
Dyslipidemia	**51 (31.3)**	**35 (21.3)**	**0.045**	3 (9.4)	8 (12.1)	0.752
Hepatic steatosis	56 (34.4)	50 (30.5)	0.292	4 (12.5)	3 (4.5)	0.211
Hepatic cirrhosis	4 (2.5)	3 (1.8)	0.723	0 (0.0)	0 (0.0)	1.000
Other hepatic diseases[d]	5 (3.1)	9 (5.5)	0.414	1 (3.1)	1 (1.5)	0.549
Diabetes mellitus	**27 (16.6)**	**13 (7.9)**	**0.019**	2 (6.3)	2 (3.0)	0.595
Chronic kidney disease[e]	13 (8.0)	7 (4.3)	0.165	2 (6.3)	2 (3.0)	0.600
COPD / emphysema	**23 (14.1)**	**8 (4.9)**	**0.005**	2 (6.3)	5 (7.6)	1.000
Asthma	17 (10.4)	12 (7.3)	0.338	14 (43.8)	27 (40.9)	0.830
Allergic contact dermatitis	15 (9.2)	13 (7.9)	0.698	14 (43.8)	24 (36.4)	0.513
Allergic rhinoconjunctivities	24 (14.7)	28 (17.1)	0.651	20 (62.5)	41 (62.1)	1.000
Food allergies	12 (7.4)	15 (9.1)	0.689	14 (43.8)	33 (50.0)	0.667
Depression	42 (25.8)	28 (17.1)	0.060	6 (18.8)	10 (15.2)	0.772
Neoplastic diseases[f]	8 (4.9)	3 (1.8)	0.070	0 (0.0)	4 (6.1)	1.000
**Smoking status**						
Current smoker	49 (30.1)	63 (38.4)	0.111	6 (18.8)	25 (37.9)	0.056
Ex-smoker	**83 (50.9)**	**61 (37.2)**	**0.014**	12 (37.5)	15 (22.7)	0.232
Never smoker	31 (19.0)	40 (24.4)	0.423	14 (43.8)	26 (39.4)	0.082
**Previous infectious diseases**						
Pneumonia	34 (20.9)	22 (13.4)	0.136	4 (12.5)	14 (21.2)	0.404
Years since pneumonia, mean (SD)	16.8 (16.2)	14.0 (14.3)	0.509	14.8 (9.5)	14.8 (8.5)	0.997
Hospitalization due to pneumonia[g]	10 (29.4)	4 (18.2)	0.343	1 (25.0)	6 (42.9)	0.518
Bronchitis	**57 (35.0)**	**37 (22.6)**	**0.042**	13 (40.6)	16 (24.2)	0.230
Years since bronchitis	7.6 (10.0)	7.8 (9.6)	0.949	8.5 (11.6)	3.8 (4.1)	0.197
Hospitalization due to bronchitis[g]	**8 (14.0)**	**0 (0.0)**	**0.020**	1 (7.7)	0 (0.0)	0.448
Herpes zoster	32 (19.6)	24 (14.6)	0.242	5 (15.6)	8 (12.1)	0.754

Years since Herpes zoster, mean (SD)	15.4 (14.6)	14.0 (11.8)	0.694	13.4 (15.4)	13.9 (12.4)	0.955
(Post)herpetic neuralgia[g]	26 (81.3)	17 (70.8)	0.361	4 (80.0)	7 (87.5)	0.715
**Disease activity**						
PASI median (IQR)	1.9 (0.0 -4.0)	1.6 (0.6-4.3)	0.562	-	-	-
EASI median (IQR)	-	-	-	9.6 (2.3-17.2)	11.4 (3.2-17.4)	0.575
DLQI median (IQR)	2.0 (0.0-8.0)	3.0 (1.0-9.0)	0.347	11.5 (5.0-18.0)	13.0 (4.3-18.0)	0.704
Current immunosuppressive therapy[h]	137 (84.0)	137 (83.5)	0.900	16 (50.0)	26 (39.4)	0.320

Table S3. Regression analyses predicting reasons against influenza vaccination in PsO patients. The probability of stating a distinct reason against vaccination (shown in the heading) was chosen as the dependent variable. Age, gender, occupational status, and PsA served as independent variables. Significant predictors are printed in bold. a The reference category to female was male. b The reference category of working full-time was any other occupational status. The model was adjusted for the center of recruitment (not shown). SE: standard error.

(a) Vaccination not deemed necessary by patient.

Predictor	Coefficient (SE)	p-value
Age	0.003 (0.003)	0.302
Female[a]	-0.026 (0.086)	0.759
Working full-time[b]	-0.022 (0.085)	0.797
PsA	-0.010 (0.082)	0.902
**Constant**	**0.475 (0.235)**	**0.045**

(b) No personal history of severe flu.

Predictor	Coefficient (SE)	p-value
Age	0.001 (0.003)	0.635
Female[a]	0.109 (0.086)	0.204
Working full-time[b]	0.113 (0.083)	0.176
PsA	-0.029 (0.081)	0.721
Constant	0.358 (0.240)	0.137

(c) Lacking recommendation by a physician.

Predictor	Coefficient (SE)	p-value
**Age**	**-0.006 (0.003)**	**0.038**
Female[a]	-0.118 (0.080)	0.141
Working full-time[b]	0.104 (0.083)	0.209
PsA	-0.029 (0.077)	0.713
**Constant**	**0.471 (0.237)**	**0.049**

(d) Lacking confidence in protective effect.

Predictor	Coefficient (SE)	p-value
Age	0.002 (0.003)	0.452
Female[a]	0.012 (0.077)	0.875
Working full-time[b]	-0.038 (0.075)	0.615
PsA	-0.067 (0.069)	0.338
Constant	0.202 (0.190)	0.290

(e) Potential side effects.

Predictor	Coefficient (SE)	p-value
Age	0.002 (0.002)	0.500
Female[a]	0.018 (0.073)	0.806
Working full-time[b]	0.121 (0.073)	0.099
PsA	-0.011 (0.070)	0.872
Constant	0.175 (0.185)	0.346

(f) Patient forgot to get vaccinated.

Predictor	Coefficient (SE)	p-value
Age	-0.002 (0.002)	0.349
Female[a]	-0.030 (0.045)	0.509
Working full-time[b]	0.052 (0.050)	0.292
PsA	0.039 (0.049)	0.432
Constant	0.139 (0.131)	0.290

(g) No time for vaccination.

Predictor	Coefficient (SE)	p-value
Age	-0.001 (0.002)	0.539
Female[a]	0.007 (0.048)	0.879
**Working full-time[b]**	**0.135 (0.049)**	**0.006**
PsA	-0.033 (0.053)	0.674
Constant	0.167 (0.119)	0.161
